# Vision and Hearing Impairments in Relation to Disability-Free Life Expectancy in People From England and the United States

**DOI:** 10.1093/geroni/igaa057.1843

**Published:** 2020-12-16

**Authors:** Paola Zaninotto, Giorgio Di Gessa, Jenny Head

**Affiliations:** 1 University College London, London, United Kingdom; 2 University College London, London, England, United Kingdom; 3 UCL, London, England, United Kingdom

## Abstract

Both hearing and vision impairments are some of the most common deficits experienced by older adults. We examined the impact of self-reported vision and hearing impairments on disability-free life expectancy (DFLE). We used harmonized data from the Gateway to Global Aging Data from the US Health and Retirement Study (HRS) and the English Longitudinal Study of Ageing (ELSA). We used discrete-time multistate life table models to estimate disability-free life expectancy by sex, age and country. In both countries and at all ages either vision or hearing impairment was associated with shorter DFLE compared to those who reported no impairments. Reporting both vision and hearing impairments reduced DFLE. For example, at the age of 50, men and women with both vision and hearing impairments could expect to live up to 12 fewer years free from disability compared with men and women with no impairments, similar results were found in both countries.

